# Establishment of an indirect ELISA for detection of the novel antifibrotic peptide M10

**DOI:** 10.1371/journal.pone.0188588

**Published:** 2017-11-27

**Authors:** Tanjina Akter, Ilia Atanelishvili, Atsushi Noguchi, Richard M. Silver, Galina S. Bogatkevich

**Affiliations:** 1 Division of Rheumatology and Immunology, Department of Medicine, Medical University of South Carolina, Charleston, South Carolina, United States of America; 2 Division of Rheumatology, Endocrinology and Nephrology, Hokkaido University Graduate School of Medicine, Sapporo, Japan; Pennsylvania State Hershey College of Medicine, UNITED STATES

## Abstract

**Objective:**

M10 is a ten amino acid peptide generated from the intracellular cytoplasmic tail of the hepatocyte growth factor (HGF) receptor c-Met following cleavage by caspase-3. Recently we reported that M10 interacts with Smad2 and demonstrates antifibrotic properties in vitro and in vivo and can be advanced into a novel antifibrotic remedy. The current study was undertaken to develop an immunoassay to measure M10 concentration in biological specimens.

**Experimental design:**

An Indirect Enzyme-Linked Immunosorbent Assay (ELISA) for detection of M10 in biological fluids was developed using pharmaceutical grade synthetic M10 as a calibrator and commercially available anti-c-Met C12 antibody.

**Results:**

M10 ELISA specifically detected in plasma M10, but not a scrambled peptide, following a single intraperitoneal administration of M10 (1mg/kg) to mice. The detection limit was 9.6 ng/ml, and the measuring limit was between 15 ng/ml and 200 ng/ml. The recovery limits of M10 were between 80% and 120%; intra-assay coefficient of variation was between 5.3% and 6.3%; inter-assay coefficient of variation was between 5.0% and 8.0% over the buffer concentration tested in the range from 15 ng /ml to 250 ng /ml. The peak of M10 concentration following a single intraperitoneal injection (1mg/kg) was achieved within 6 hours and declined to minimal levels by 48 hours. The experimentally obtained half-life for M10 was comparable to the theoretically predicted half-life for M10.

**Conclusions:**

We have established a highly sensitive ELISA to detect the antifibrotic peptide M10 in plasma samples, which should prove to be a novel tool to study the pharmacokinetics and efficacy of M10 in the treatment of fibroproliferative disorders.

## Introduction

Nearly 45% of all deaths in the developed world are attributed to some type of chronic fibroproliferative disease [1]. Most of the fibrotic diseases, such as idiopathic pulmonary fibrosis (IPF) and scleroderma associated interstitial lung disease (SSc-ILD), have a poor prognosis that is comparable to end-stage cancer. Although pirfenidone and nintedanib, each approved by the Food and Drug Administration (FDA) in 2014, may slow the rate of decline of lung function in some IPF patients, neither drug significantly alters the course of this lethal disease [2–4].

We recently discovered a 10 amino acid antifibrotic peptide (M10) with antifibrotic properties in vitro and in a murine model of lung fibrosis, which is a natural cleavage product by caspase-3 of the cytoplasmic tail of the c-MET receptor tyrosine kinase [5, 6]. Following binding of hepatocyte growth factor (HGF), c-MET undergoes auto phosphorylation at tyrosine residues in its cytoplasmic domain and initiates a cascade of signal transduction events leading to specific cellular responses implicated in embryonic development and tissue regeneration after injury [7–10]. Interestingly, c-MET appears in all vertebrates tracing back to a single protochordate ancestor [11]. However, M10 has appeared only in higher primates (humans and great apes) but not in any other mammals [12]. We demonstrated that M10 interacts with Smad2 and reduces collagen in the bleomycin-induced mouse model of lung fibrosis and in human lung and skin fibroblasts [5]. Our data suggest that M10 might be advanced into an efficacious therapy of SSc-ILD, IPF, or other fibrosing diseases.

During the past decade, peptides have gained a wide range of applications in medicine and biotechnology, and therapeutic peptide research is also currently experiencing a renaissance for commercial reasons [13]. Peptides are recognized for being highly selective efficacious medications that offer potential of low dose administration without major side effects. However, lack of the oral bioavailability, poor stability and relatively short circulating plasma half-life serve as significant challenges for peptide-based drug candidates [14]. One of the potential strategies for improving the effectiveness of peptide-based drug development is to obtain pharmacokinetic (PK) data on early stages in order to access more accurately the potential for dug efficacy [15].

In this manuscript we present a highly sensitive indirect enzyme-linked immunosorbent assay (ELISA) that was developed using synthetic M10 as an antigen. Next, we successfully used the M10 ELISA to quantify M10 concentrations in plasma samples in order to obtain the pharmacokinetic profile of the peptide.

## Materials and methods

### Materials

Peptides (M10 and scrambled control peptide) were obtained from GenScript (Piscataway, NJ); anti-Met (C12) antibody was purchased from Santa Cruz Biotechnology (Santa Cruz, CA) and HRP-conjugated anti-Rabbit IgG antibody was purchased from Rockland (Limerick, PA). High-affinity polystyrene micro-titer plates were obtained from VWR (Radnor, PA); ultra-3, 3’, 5, 5’-tetramethylbenzidine (TMB)-substrate was obtained from Thermoscientific (Waltham, MA).

### Sample preparation

The study was approved by the Institutional Animal Care and Use Committee of the Medical University of South Carolina. Mice (n = 30), C57BL/6 males and females 8–10 weeks old weighing 20–25 g, purchased from Jackson Laboratories (Bar Harbor, ME) were used in this study. Mice were housed in ventilated racks in specific pathogen free conditions and were provided with food and water available *ad libitum*. M10 peptide was solubilized in water and administered intraperitoneally in a dose of 1 mg/kg. Mice were sacrificed by isoflurane overdose, and blood samples were collected by cardiac puncture in the presence of 0.01% of Na-Citrate at time intervals of 15 min, 30 min, 1, 2, 4, 6, 12, 24, and 48 hours after injection. The blood samples (n = 3/group) were centrifuged at 2500 rpm for 10 min, and plasma samples were stored at -80°C. Plasma samples collected from mice injected with water intraperitoneally (n = 3) were used as a negative control.

### General protocol of enzyme-linked immunosorbent assay

M10 concentration was measured by indirect ELISA developed in our laboratory. Known concentrations of pharmaceutical grade M10 peptide (GenScript, Piscataway, NJ) with purity more than 98% were used to generate the standard curve. Briefly, 100μl of known concentration of M10 (range: 10 ng/ml to 500 ng/ml), blank (PBS without M10), and 100μl of the diluted plasma sample were plated in triplicate in high-affinity polystyrene micro-titer plates. Samples were incubated with gentle rocking overnight at 4°C and blocked with 5% bovine serum albumin in PBS. After washing, samples were incubated for 2 hours at room temperature with anti-cMET rabbit antibody (Santa Cruz Biotechnology, Santa Cruz, CA), followed by 1hour incubation with HRP-conjugated anti-Rabbit IgG (Rockland, Limerick, PA). M10 was detected with ultra-TMB (3, 3’, 5, 5’-tetramethylbenzidine)-substrate, and the reaction was terminated by using 2M sulfuric acid. Absorbance was measured at 450 nm by Spectra Max Plus spectrophotometer. Concentrations of unknown samples were then calculated from their corresponding optical density (OD) values by Softmax Pro 4.8 software (Molecular Devices, Sunnyvale, CA) using the standard curve generated from the known concentrations of M10. Control plasma samples collected from mice with intraperitoneal water injection were run in triplicate with each experiment, mean was calculated, and samples were normalized by subtracting the negative control mean value. Final concentrations were calculated by adjusting the sample dilution factor.

### Calibrators, controls, and standard curve development

Samples, controls and standards were allowed to bind to high affinity polystyrene plates in a temperature-controlled environment. A variety of polystyrene plates including Immulon 2HB, Immulon 4 HBX, 100–200 ng of Ig/cm2 capacity medium binding plate, and 400–500 ng of Ig/cm2 capacity high binding plate with or without pretreatment of 3-aminopropyl-thiethoxysilane were tested. Different solvents such as H_2_O, PBS (pH 7.0) or sodium bicarbonate buffer (pH 9.6) were used as a coating buffer to prepare the calibrators, and then the most favorable condition for hydrophobic interaction was evaluated. To eliminate the batch effects of the capture and detection antibodies, all experiments were conducted by using the same lot of antibodies. A series of calibrators were prepared by dissolving a known concentration of M10 in PBS followed by serial dilutions. PBS without M10 was used as a blank, considered as 0.0 ng/ml concentration to generate the standard curve. Plasma, collected from mice that received intraperitoneal H_2_O was used as a negative control. Negative control plasma samples spiked with known concentrations of M10 were used as a positive control. Mouse plasma samples collected at distinct time intervals following intraperitoneal M10 injection were aliquoted, stored at –80°C and used as samples. On the day of analysis, a standard curve was generated by plotting the concentration and corresponding absorbance measured at 450 nm. All measurement of controls, samples and calibrators were tested in triplicate. Any samples with high percentage of intra-assay absorbance variability were eliminated from analysis.

### Determination of optimal antibody concentration

Antibody working concentrations were determined by titrating both primary and secondary antibodies. A series of diluted primary antibody were tested against different conditions of detection antibody and inherent reactivity was evaluated. The best combination of primary and secondary antibodies was determined by estimating the coefficient of determination (R2) in four-parametric-logistic regression model by using SoftMaxPro (V4.8). ELISA conditions that achieved R2>0.95 were considered for further optimization (Table 1). The correlation between concentration and absorbance was verified with Pearson correlation, data with significant (p< 0.05) coefficient was used for analysis.

**Table 1:** Coefficient of determination (R^2^) values corresponding to antibodies, concentrations and condition.

Antibody	Dilution	R^2^ value	Antibody	Dilution/condition	R^2^ value
Primary	1:500	0.97	Secondary	1: 10,000	0.99
	1:600	0.99		1:20,000	0.96
	1:700	0.97		1:20,000 + 2.5% BSA	0.97
	1:800	0.97		1:20,000 + 5% BSA	0.98
	1:1000	0.98			

### Specificity

To test the specificity of this assay, mouse plasma samples were collected following intraperitoneal injection of scrambled peptide. To verify the biological activity of the scrambled peptide, different forms of scrambled peptides were designed and the antifibrotic properties were tested in *in-vitro* experiments. A scrambled peptide with no biological effects was used in *in- vivo* experiments. Plasma collection and storage procedures were followed as described above. Samples were diluted in PBS with different dilution factors, such as 20, 40 or 60 to make it fit within the range of the standard curves. The cross-reactivity was also tested by using control mouse plasma spiked with known concentrations of scrambled peptide. In each of these cases, the scrambled peptide generated signals below the detectable range. In M10 ELISA, the limit of detection (LOD) / minimum detectable concentration (MDC) was calculated by analyzing the mean of blank sample values from fifteen wells, in addition with 3 times of Standard Deviation (mean Blank +3*SD) [16]. PBS was run in triplicate as a blank sample in five independent ELISAs, the mean value was calculated and used for LOD estimation.

### Analytical validation

To estimate the lowest detectable M10 concentration, a series of low concentrations of M10 from 1ng/ml to 15 ng/ml in plasma were tested. M10 concentration that generated the highest absorbance above the background signal with a sufficient signal to noise ratio was used as the lowest point to develop the standard curve. Reproducibility between the wells was tested by measuring the intra-assay variance. Three different known concentrations of M10 samples (high, medium and low) were run in 24 replicates, and the intra-assay coefficient of variation was calculated. The inter-assay precision was measured by testing known M10 samples (high, medium and low) in triplicate on four subsequent independent assays. To measure the analytical recovery, buffer spiked with known concentration of M10 was four times serially diluted. Each of these concentrations was considered as an individual sample and was run in triplicate. The analytical recoveries were calculated by comparing the estimated concentrations with the expected concentrations and are presented in percentage.

### Statistical analysis

Statistical analysis of data was performed using Softmax Pro 4.8 software (Molecular Devices, Sunnyvale, CA), KaleidaGraph 4.0 (Synergy Software, Reading, PA), and GraphPad Prism 7 statistical software (GraphPad Software, Inc. La Jolla, CA).

## Results

### Development of the M10 ELISA

**Fig 1:**
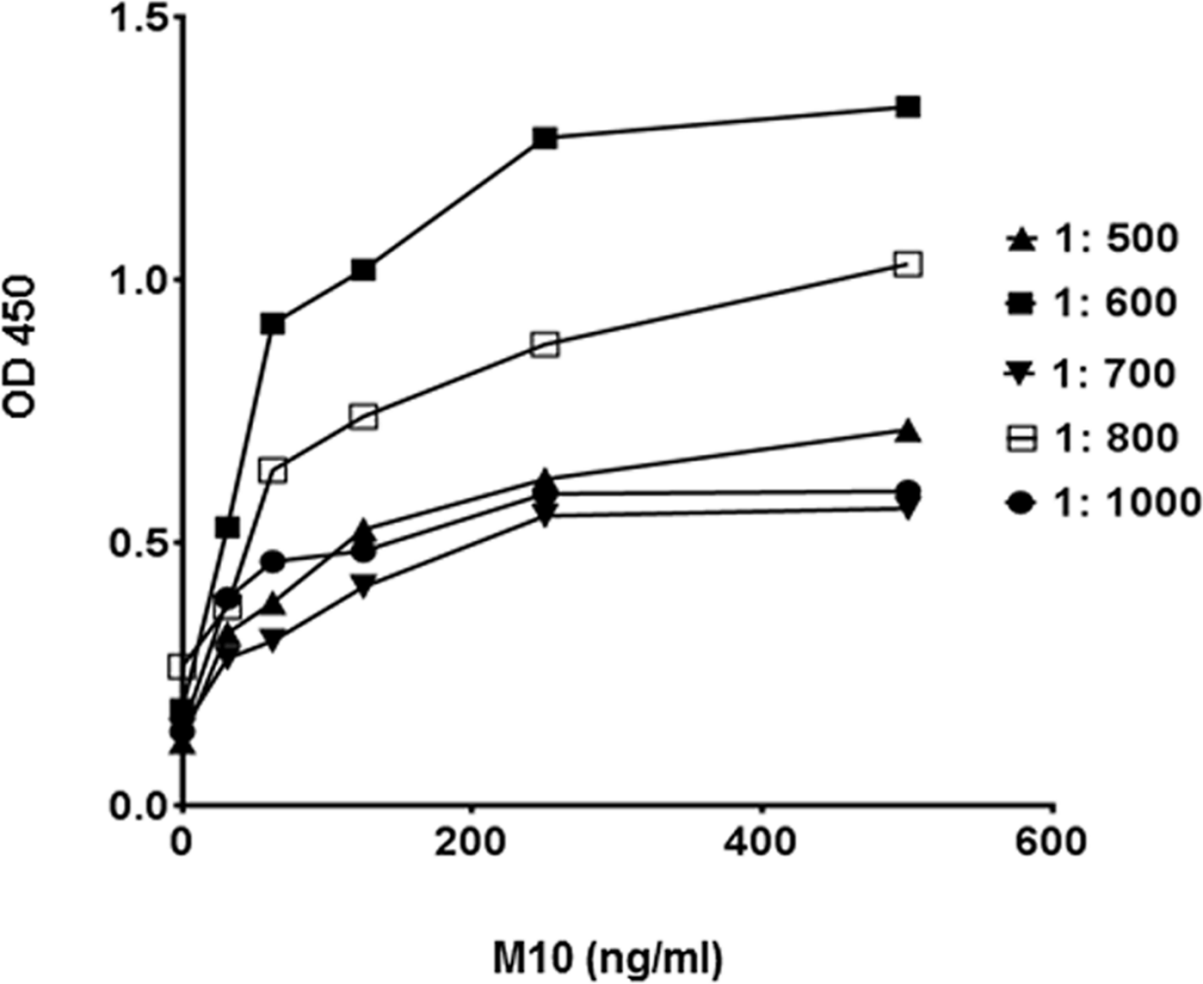
Effects of primary antibody concentrations on sensitivity in M10 ELISA. A series of primary antibody dilutions (1:500, 1:600, 1:700, 1:800, 1:900 and 1:1000) in PBS were tested against 1:10,000 (0.2 μg /ml) concentration of detection antibody. Each data point represents the mean value of the corresponding triplicates.

**Fig 2:**
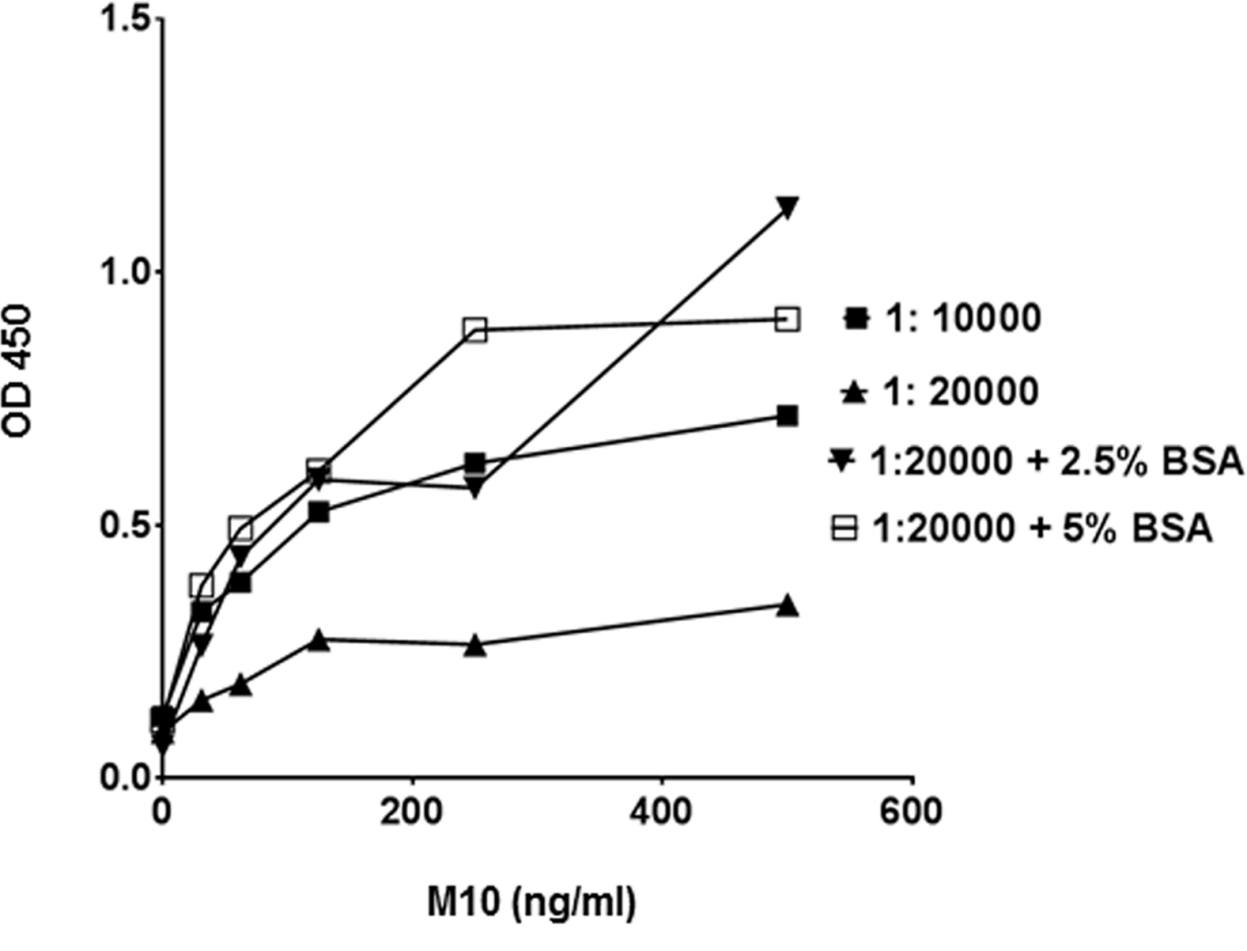
Effects of detection antibody conditions on sensitivity in M10 ELISA. Detection antibody at a dilution of 1:10,000; 1:20,000; 1: 20,000 in presence of 2.5% bovine serum albumin and 1: 20,000 in presence of 5.0% bovine serum albumin in PBS, were tested against 1: 600 (0.3 μg / ml of M10) concentration of primary antibody. Each data point represents the mean value of the corresponding triplicates.

To facilitate the direct adsorption of M10 to the assay plate a number of conditions including a variety of polystyrene plates, coating buffers, and incubation temperatures were tested. We observed that M10 dissolved in PBS provides the best hydrophobic interaction with 400–500 ng of Ig/cm2 capacity high binding polystyrene plates. Next, we optimized antibody pair concentrations while keeping the above-described conditions constant. Primary antibody was serially diluted from 200μg/ml to 200ng/ml, whereas the concentration of HRP-conjugated secondary anti-rabbit antibody remained constant 200ng/ml (Fig 1). Subsequently, different concentrations and conditions of secondary antibody were tested to develop the highest sensitivity and lowest background signals (Fig 2). Depending on the inherent reactivity properties, 1: 600 (0.3 μg/ml) dilution of primary antibody and 1:20,000 + 5% BSA (100ng/ml antibody in 5% BSA) were selected for the standard assay protocol.

**Fig 3:**
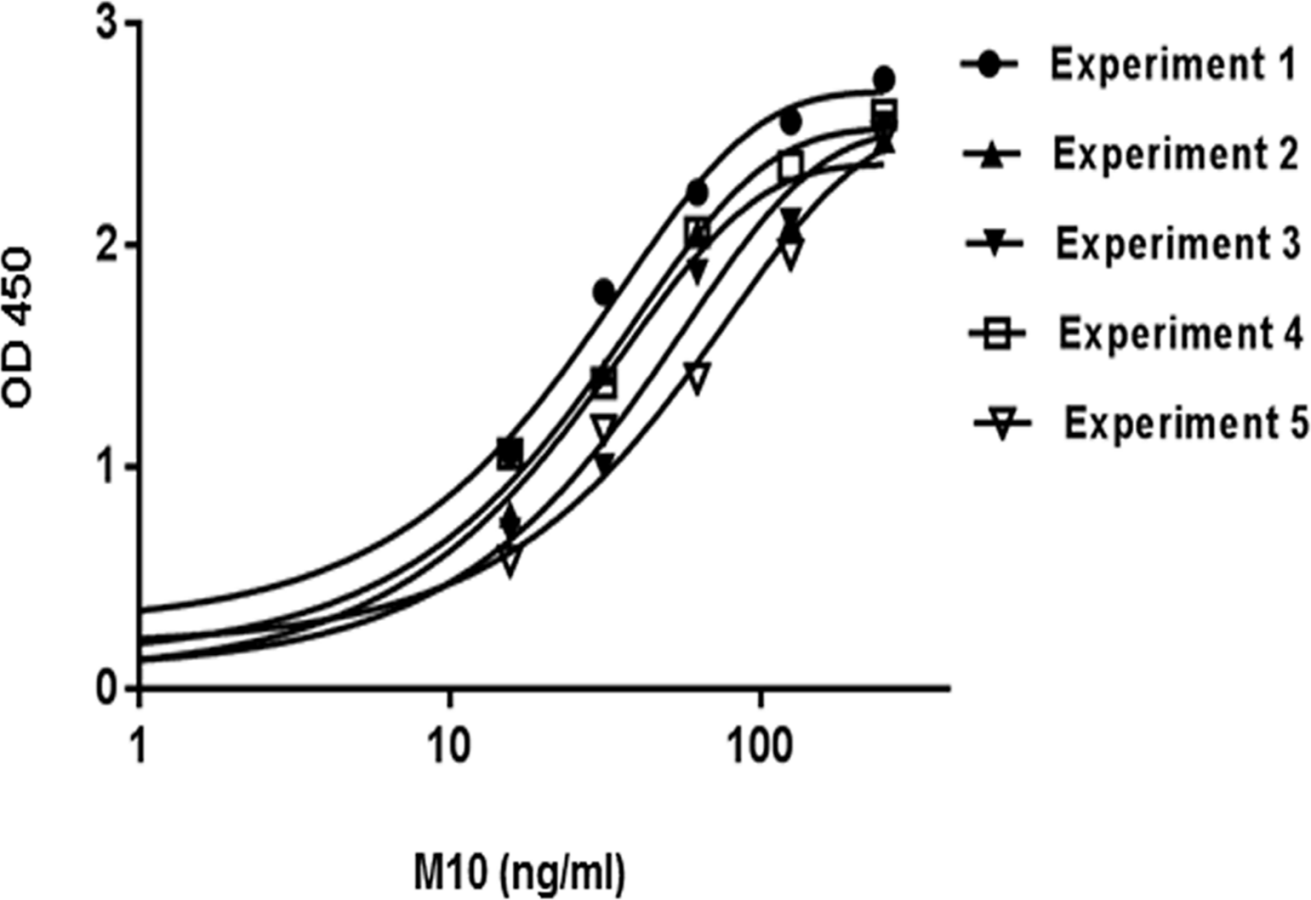
The calibration curves of M10. Individual lines represent independent experiment and each data point represents the mean value of the corresponding triplicates. Absorbance of standards was measured at 450 nm and graph was generated by using 4-parametric logistic regression model.

Blocking buffer conditions, incubation times and incubation temperatures were determined based on the experimental data characterized by the minimal background signals. Known concentrations of pharmaceutical grade M10 were serially diluted from 250ng/ml to 15.6ng/ml with PBS and measured in triplicate to generate standard curves. A plot of serial dilutions of M10 standard revealed a sigmoidal-type curve typical for most antibody-antigen interactions (Fig 3). The standard curves obtained from five independent experiments and fitted in four-parametric logistic regression models demonstrate the reproducibility of this assay.

The specificity of the M10 ELISA was tested by using scrambled M10 peptide as an antigen in the test sample. The scrambled peptide was diluted in three different concentrations (200ng/ml, 100ng/ml and 50ng/ml) and tested in the M10 ELISA. These test samples either had no signal or generated signals below background level.

### Detection limit, accuracy, and recovery of the assay

Samples representing low and high concentrations of M10 were used to calculate coefficients of variation within and between assays. The minimum detection limit of the assay was estimated by measuring a series of low concentrations of M10 between 0ng/ml and 15ng/ml. The lowest concentration to generate signal above background was 15ng/ml. Each of the concentrations was run in triplicate and three independent experiments were performed. Method validation and quality test of the M10 ELISA were performed by intra-assay precision, inter-assay precision and by recovery estimation. Three different known concentrations of M10 calibrators (200ng/ml, 100ng/ml and 50ng/ml) were measured in twenty-four replicates in one assay. The co-efficient of variance (CV) of low-, medium- and high-concentration samples was 6.3%, 4.0%, and 5.2%, respectively, indicating excellent intra-assay repeatability (Table 2).

**Table 2:** Precision of the M10 ELISA.

Sample	Calibrator (ng/ml)	Range	CV^a^ (%)
**Intra-assay**			
Low	50	53.0 ± 3.36	6.33
Medium	100	93.9 ± 3.08	4.04
High	200	238.5 ± 12.5	5.25
**Inter-Assay**			
Low	50	51.9 ± 2.59	5.0
Medium	100	100.3 ± 8.02	8.01
High	200	224.6 ± 14.68	6.54

Range values are the mean and standard deviation from five independent experiments

^a^CV stands for co-efficient of variance

To estimate the inter-assays CV, three known calibrators (200ng/ml, 100ng/ml and 50ng/ml) of M10 were tested in triplicate in four independent experiments. The CV for inter-assays at three different levels of M10 was 5.0%, 8.01%, and 6.5% (Table 2). To eliminate the batch effect, all experiments were performed with the same lot of antibodies.

To determine the antigen recovery, 250ng/ml of M10 was serially diluted at a ratio of 1:1; 1:2; 1:4; 1:8 and 1: 16. Each of these dilutions was considered as an individual sample and run in triplicate over five independent experiments. The analytical recoveries, presented as the estimated concentrations compared with the expected concentrations, ranged between 89.9% and 112.9% (Table 3).

**Table 3:** Analytical recovery of M10.

Dilution	Calibrator (ng/ml)	% of Recovery
1:1	250	105.96 ± 2.8
1:2	125	101.56 ± 6.9
1:4	62.5	95.2 ± 2.4
1:8	31.25	112.9 ± 7.6
1:16	15.6	89.9 ± 7.3

% of Recovery values are the mean and standard error from 5 independent experiments

The calculated LOD of M10 Elisa 9.6 ng/ml was estimated according to the previous stated method. The estimated assay range was between15.6 ng/ml and 200.0 ng/ml; the evaluation was based on certain criteria such as, recovery rate, reproducibility and percent of variation (Table 4).

**Table 4:** Specification of M10 ELISA.

Detection Method	Limit of Detection	Assay range	Detector antibody conjugate
Colorimetric	9.6 ng/ml	(15.6–200) ng/ml	HRP/TMB

### Plasma concentrations of M10

**Fig 4:**
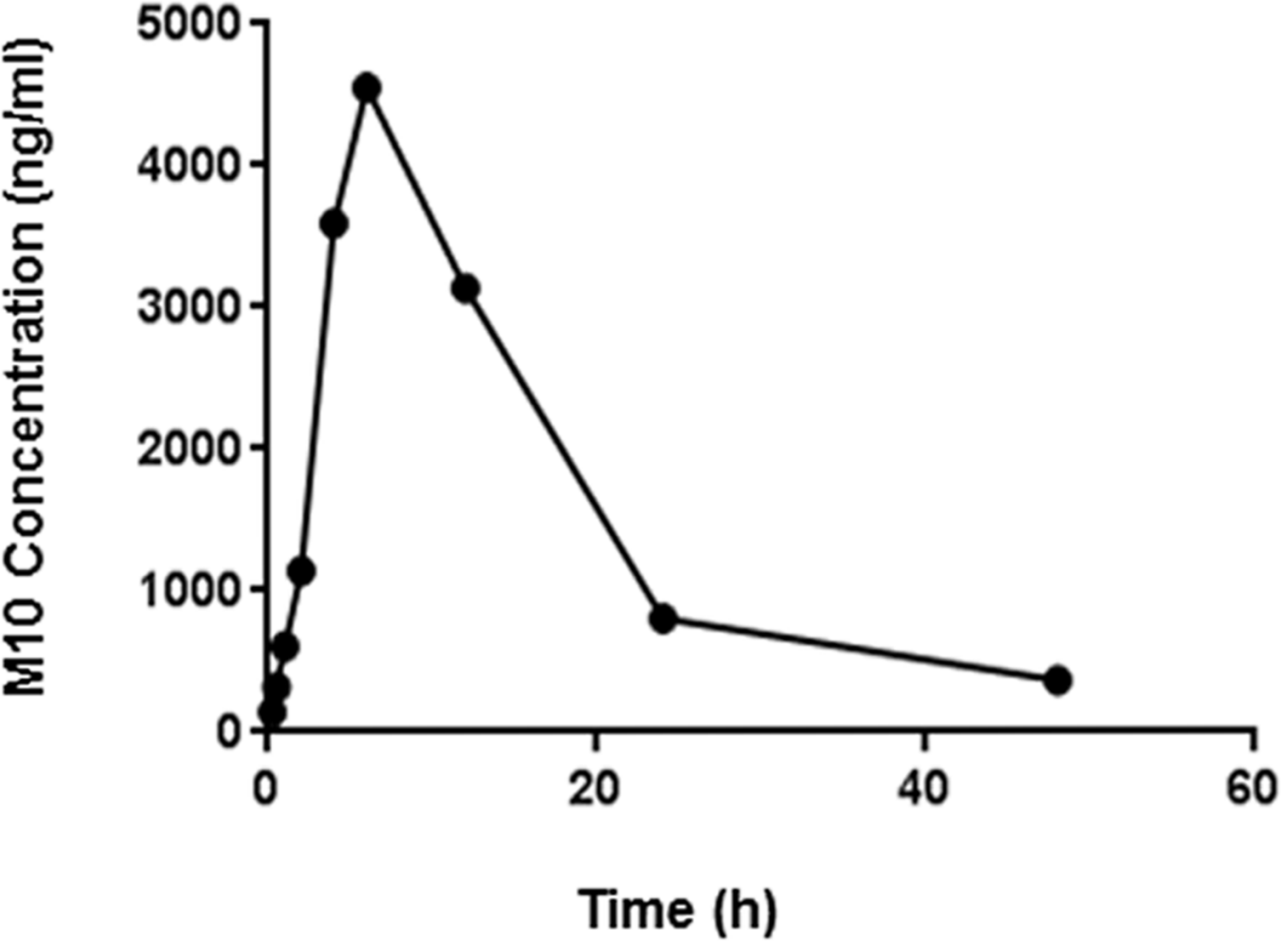
Pharmacokinetic profile of M10 in mouse plasma. Plasma was collected over a period of 15 minutes—48 hours after M10 injection and measured by indirect M10 ELISA. Plot represents the experimentally estimated M10 concentration over time course to model prediction following IP injection in mice.

To obtain the pharmacokinetic profile of M10 in mouse plasma, C57Bl6 mice were injected intraperitoneally with M10 at a dose of 1mg/kg of body weight. Plasma samples were collected by cardiac puncture in the presence of 0.01% of Na-Citrate at time intervals of 15 min, 30 min, 1h, 2h, 4h, 6h, 12 h, 24 h, and 48 h following injection. Samples were diluted in PBS in order to fit within the range of the standard curves and then tested using the M10 ELISA. Concentration of M10 in plasma samples was calculated by Softmax Pro 4.8 software using the standard curve generated from the known concentration of M10. The samples were normalized by subtracting the negative controls represented by plasma samples obtained from mice injected with H_2_O. We observed that a peak M10 concentration was achieved within 6 h and then declined to minimal detectable levels by 48 h (Fig 4). The area under the curve (AUC 0-48h), plasma half-life (t_½_), peak plasma concentration (Cmax) and time to reach the peak plasma concentration (Tmax) of M10 are shown as absolute values in Table 5. The half-life was determined following the method of residuals according to the equation, N_t_ = N_0_(1/2)t/t _½_; where N_0_ is the initial quantity of M10, N_t_ is the quantity of M10 that still remains after 48 h following of M10 injection, and t is a time point of 48 h [17–19].

**Table 5:** The pharmacokinetic parameters of M10.

PK parameters	Values
Dose (mg/ kg of mouse body weight)	1.0
AUC 0-48h (ng.h/ml)	72463 ± 2390
Half-life (h)	6.8 ± 0.7
Tmax (h)	6
Cmax (ng/ml)	4861.5 ± 11.5

The half-life, plasma concentrations for AUC and Cmax are presented with the mean ± SD. PK parameters was calculated using GraphPad Prism 7.

**Fig 5:**
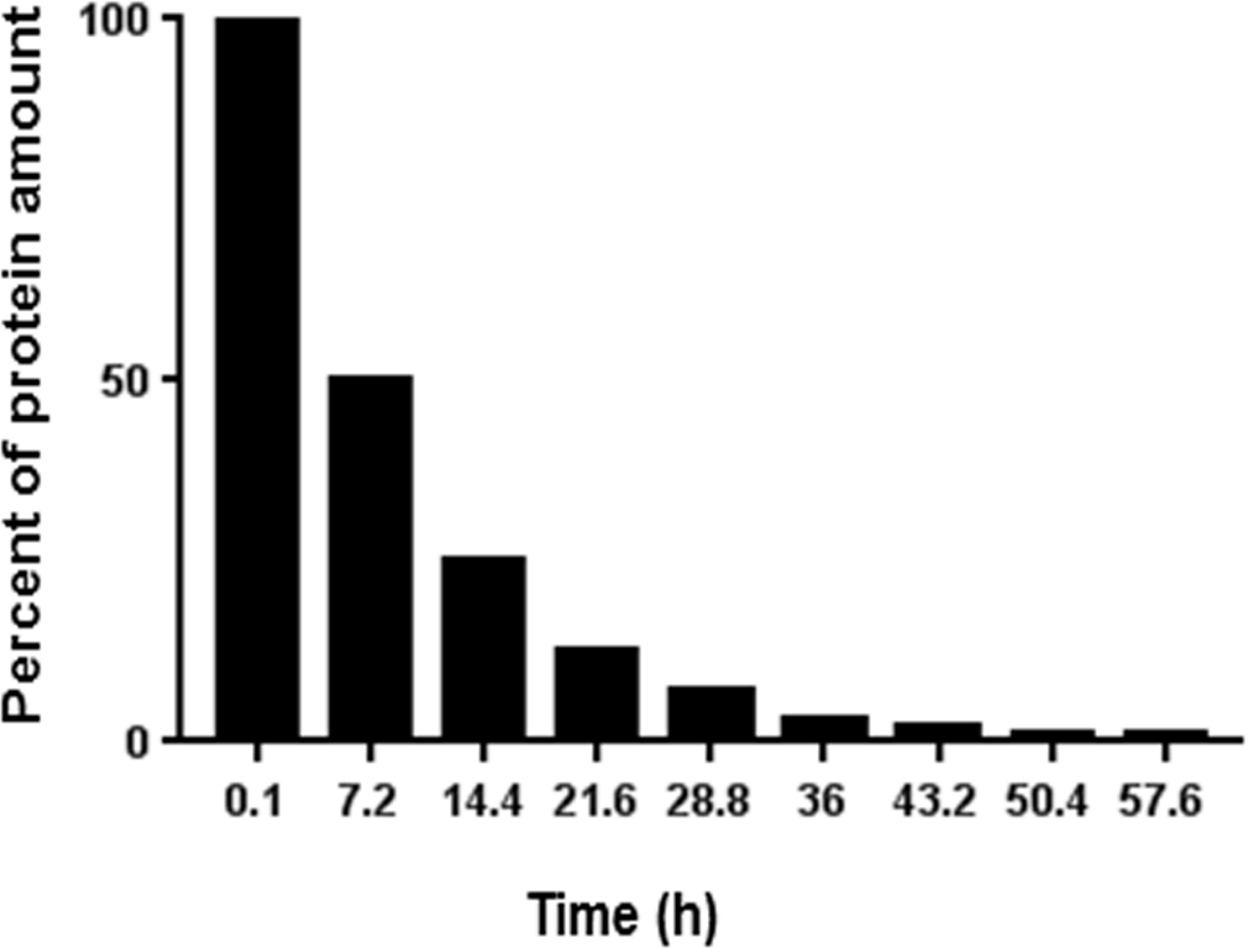
The predicted half-life of M10 in plasma was estimated by ProtLifePred software. Experimental half-life of M10 (6.8 ± 0.7 h) in mouse plasma complies with predicted half-life of M10 (7.2 h).

Using the M10 ELISA, the experimentally obtained t_½_ for M10 (6.8±0.7 h) was comparable to the theoretically predicted t _½_ for M10 of 7.2h by ProtLifePred software [20, 21] (Fig 5).

## Discussion

With the increase of approved peptide-based drugs and the advances in peptide-associated technologies, peptide-based therapeutics open up novel opportunities for treating human diseases. Over the past decade, thousands of new peptides as highly potent signal transduction molecules have been identified. Given their attractive pharmacological profile and intrinsic properties, many peptides, especially small and long acting, have gained a wide range of interest as potential drug candidates [22, 23].

We have discovered a ten amino acid peptide, “M10”, which is derived from the C-terminal part of the MET receptor tyrosine kinase via naturally occurring caspase-3 mediated cleavage [5, 6]. We showed that M10 demonstrates profound antifibrotic effects in the bleomycin-induced mouse model of pulmonary fibrosis and in primary human lung and skin fibroblasts isolated from patients with scleroderma-associated pulmonary fibrosis [5]. The effective treatment for severe fibrosing diseases such as idiopathic pulmonary fibrosis and scleroderma-associated interstitial lung disease is currently lacking, which makes identification of a peptide with antifibrotic properties a potentially very exciting and important discovery. To advance M10 into an effective antifibrotic medication, a dependable method to measure the effective concentration of this peptide in order to investigate its pharmacokinetic profile is necessary. The purpose of the M10 ELISA that we have developed is to serve as a valuable tool to further characterize M10 in vitro and in vivo.

To develop an indirect ELISA for M10 quantification, we used a commercially available anti-Met C12 antibody generated against the last 12 amino acids of MET, which recognizes M10 as an antigen but does not recognize a scrambled peptide used as a control [5, 6]. We have established an ELISA method using synthetic M10 as a coating agent that yielded a sensitivity of 9.6 ng/ml and analytical recoveries between 89.9% and 112.9%. We consider the incomplete retrievals around 10% as a limitation of the M10 ELISA. The recoveries could be improved by using a more sensitive sandwich ELISA, which depends on the availability of antibodies targeted to different epitopes on protein of interest. However, M10 has only ten amino acids, rendering difficulty to generate two separate epitopes for antibody production. Therefore, a sandwich ELISA could not be developed to measure M10 concentration.

By the evolutionarily recent amplification, M10 peptide has appeared in higher primates (humans and great apes), but not in any other mammals [11, 12]. Although mice do not generate endogenic M10, synthetic M10 demonstrates intense antifibrotic effects in a mouse model of bleomycin-induced pulmonary fibrosis [5]. Previously, we showed that cleavage of MET and generation of endogenous M10 occurs in the lungs of patients with scleroderma associated pulmonary fibrosis [6]. We speculate that concentrations of endogenous M10 could be high enough to modify disease severity. The indirect M10 ELISA would be a great tool to measure endogenous M10 in patients with scleroderma associated pulmonary fibrosis.

To demonstrate the capacities of M10 ELISA in the current study, we chose to measure some of the PK characteristics of M10 in mice. Importantly, using our ELISA we found that the experimentally determined half-life for M10 is comparable to the theoretically predicted half-life of M10 peptide. The favorable half-life of M10 can be explained by its resistance to many mammalian proteases such as caspases, thrombin, factor Xa, trypsin and granzyme B [20, 21]. Thus, the analytical performance features of this ELISA indicate that it is suitable to provide comparable results for multiple studies with plasma samples.

In conclusion, an indirect ELISA method for detection of M10 reported herein is sensitive, specific, reproducible, simple, and adaptable for the analyses of plasma samples. This ELISA will serve as a valuable tool in pharmacokinetic, biodistribution, and toxicity studies of the novel antifibrotic peptide M10.

## Supporting information

S1 Raw Data Precision, analytical recovery and specification of the M10 ELISA. The Pharmacokinetic parameters of M10.
(PDF)

S2 Raw Data Coefficient of determination (R2) values corresponding to antibodies, concentrations and condition. (PDF)
