# Supplementary material for: Establishment of an indirect ELISA for detection of the novel antifibrotic peptide M10
Source: PLoS One. 2017 Nov 27;12(11):e0188588. doi: 10.1371/journal.pone.0188588 (PMC5703532; doi:10.1371/journal.pone.0188588)
Supplement: S1 Raw Data — The Pharmacokinetic parameters of M10. (PDF) [file pone.0188588.s001.pdf]

Primary antibody dilutions

M10 concentration (ng/ml)	Primary antibody dilutions, Mean OD from triplicates				
	1: 500	1:600	1: 700	1: 800	1: 1000
0	0.122	0.184	0.144	0.265	0.142
31.25	0.329	0.53	0.281	0.379	0.395
62.5	0.387	0.918	0.314	0.639	0.465
125	0.526	1.02	0.417	0.741	0.485
250	0.622	1.271	0.552	0.878	0.594
500	0.716	1.33	0.566	1.031	0.599

Secondary antibody dilutions and conditions

M10 concentration (ng/ml)	Secondary antibody dilutions, Mean OD from triplicates			
	1: 10000	1: 20000	1:20000 + 2.5% BSA	1:20000 + 5% BSA
0	0.122	0.091	0.063	0.114
15.6	0.194	0.11	0.137	0.265
31.25	0.329	0.154	0.262	0.38
62.5	0.387	0.186	0.437	0.493
125	0.526	0.274	0.59	0.607
250	0.622	0.264	0.573	0.885
500	0.716	0.343	1.125	0.907

5 independent experiments data set

	Mean OD from triplicates				
M10 concentration (ng/ml)	Experiment 1	Experiment 2	Experiment 3	Experiment 4	Experiment 5
0.01	0.3	0.1	0.09	0.1	0.14
15.6	1.07	0.79	0.71	1.06	0.58
31.25	1.79	1.43	1.00	1.38	1.17
62.5	2.24	2.06	1.88	2.06	1.4
125	2.56	2.1	2.11	2.36	1.96
250	2.75	2.48	2.55	2.59	2.49

M10 ELISA with mouse plasma
(each sample run in triplicate, 3 sample / group)

Time point	Mean M10 concentration (ng/ml)
15 min/ 0.25hr	143
30 min/0.5hr	320.6
1hr	604.4
2hr	1140
4hr	3592
6hr	4551
12hr	3135
24hr	801.2
48hr	366.4

Half life calculation of M10 by using protlifeopred software

Hour	Protein amount (%)
0.1	100
7.2	50
14.4	25
21.6	12.5
28.8	7
36	3
43.2	2
50.4	1
57.6	1

Inter-assay Table

calibrator	Read out mean of Triplicate well	Mean	std	Inter-assay variance (%)
50	55.03	51.9	2.59	5.00
	50.67			
	52.80			
	49.06			
100	94.47	100.03	8.02	8.01
	93.42			
	101.42			
	110.81			
200.0	227.60	224.60	14.68	6.54
	243.98			
	215.85			
	210.98			

Intra-assay Table

calibrator	Read out mean of Triplicate well	Mean	std	intra-assay variance (%)
200	238.8	238.52	12.53	5.25
	216.4			
	237			
	250.2			
	237.7			
	251			
100	88.7	93.94	3.80	4.05
	96.8			
	97.9			
	90.35			
	96.7			
	93.2			
50	59.7	53.05	3.36	6.34
	54.4			
	50.3			
	55.7			
	48.9			
	48.8			
	54.3			
	51.7			
	54.4			
	52.3			

Recovery Table

concentration	recovery	avg recovery	SE
15.6	110.0	89.94	7.3
	78.0		
	74.6		
	90.1		
	97.0		
31.25	112.8	112.98	7.5
	114.7		
	138.9		
	92.8		
	105.7		
62.5	101.6	95.22	2.2
	96.1		
	96.1		
	88.1		
	94.2		
125	79.5	101.56	6.9
	111.6		
	95.9		
	101.4		
	119.5		
250	100.0	105.96	2.8
	102.1		
	107.7		
	114.0		

Limit of detection (LOD)

Blank value(mean of triplicate	avg (ng/ml)	STD	LOD (ng/ml)
0.86			
5.6			
4.3	2.45	2.39	9.61
0			
1.5			

PK study data set_mouse plasma table

Time point	Mean M10 concentration (ng/ml)	
	set 1	set 2
15 min/ 0.25hr	143	451
30 min/0.5hr	320.6	763
1hr	604.4	3345
2hr	1140	4005
4hr	3592	4691.7
6hr	4551	4873.4
12hr	3135	1388.4
24hr	801.2	1216
48hr	366.4	143.4
