# Supplementary material for: Establishment of an indirect ELISA for detection of the novel antifibrotic peptide M10
Source: PLoS One. 2017 Nov 27;12(11):e0188588. doi: 10.1371/journal.pone.0188588 (PMC5703532; doi:10.1371/journal.pone.0188588)
Supplement: S2 Raw Data — (PDF) [file pone.0188588.s002.pdf]

Primary Ab: 1→500 dilution

UnknownsDilution

Sample	Wells	Values	R	Result	MeanResult	Std.Dev.	CV%	Dilution	Adj.Result

R - Outside standard range
Mean Adjusted Result:

10→1:500

Control

Sample	Wells	Sample#	Values	MeanValue

Standard Curve

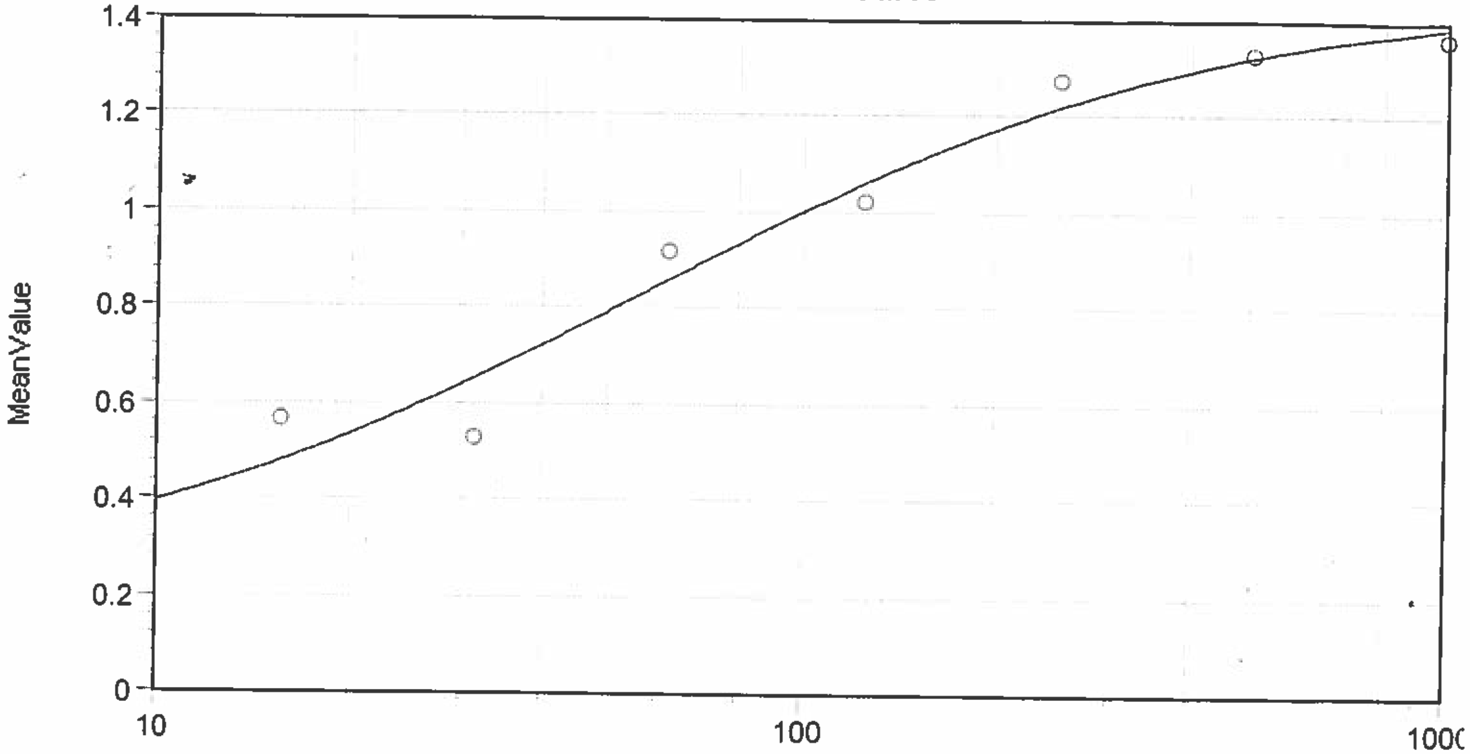

$y = ((A - D)/(1 + (x/C)^B)) + D$:	A	B	C	D	R^2
o Std (Standards: Concentration vs MeanValue)	0.199	0.975	56.492	1.459	0.976

Experiment#2

CuvetteSet#1

A1 Sa01	A2 Sa02	A3 Sa03	Endpoint
Data: No Data	Data: No Data	Data: No Data	Lm1 260
Ref: No Reference	Ref: No Reference	Ref: No Reference	Lm2 280

Copyright © 2004 Molecular Devices. All rights reserved.

Experiment#1

1:600

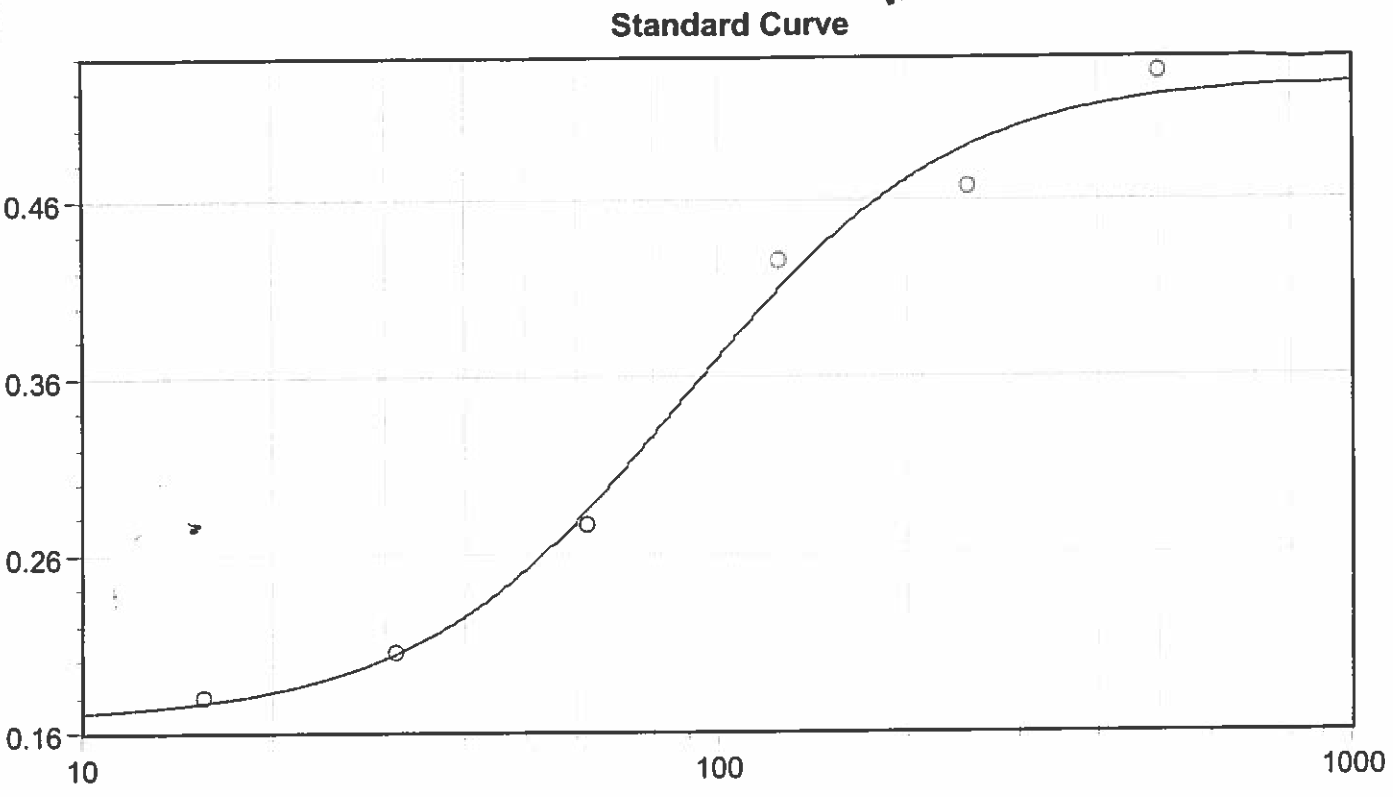

y = ((A - D)/(1 + (x/C)^B)) + D:

	A	B	C	D	R^2
o Std (Standards: Concentration vs MeanValue)	0.167	2.083	88.777	0.528	0.992

Copyright © 2004 Molecular Devices. All rights reserved.

1°Ab = 1:700

Primary Ab : 1 → 100 dilution

Standard Curve

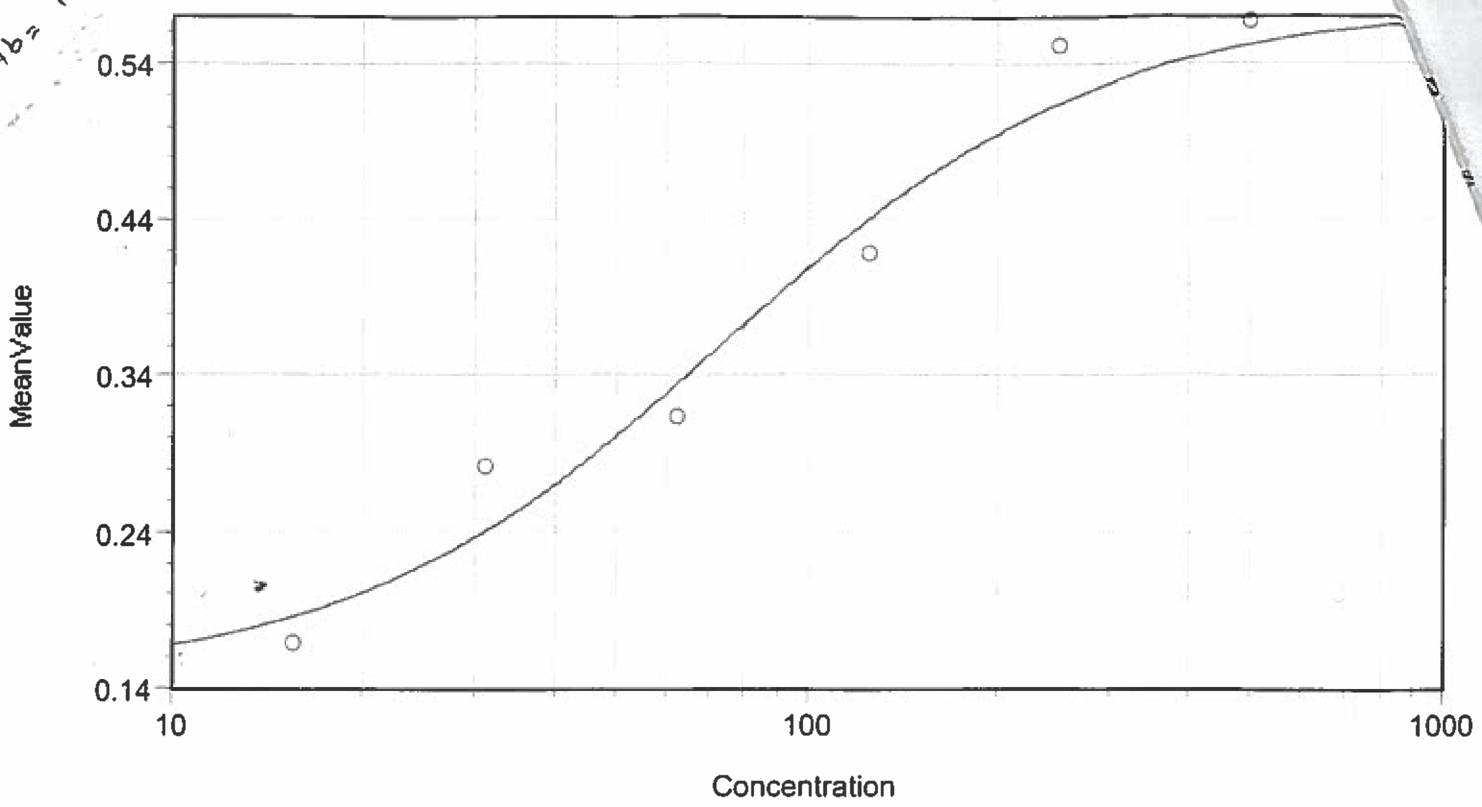

	y = ((A - D)/(1 + (x/C)^B)) + D:	A	B	C	D	R^2
o	Std (Standards: Concentration vs MeanValue)	0.147	1.477	73.916	0.576	0.974

Experiment#2

CuvetteSet#1

A1 Sa01	A2 Sa02	A3 Sa03	Endpoint
Data: No Data<br>Ref: No Reference	Data: No Data<br>Ref: No Reference	Data: No Data<br>Ref: No Reference	Lm1 260<br>Lm2 280

Wavelength Combination: !Lm1
Data Mode: Absorbance

Samples

Sample	Wells	Sample#	OD260	OD280
**Sa01**	A1	1		
**Sa02**	A2	2		
**Sa03**	A3	3		

Copyright © 2004 Molecular Devices. All rights reserved.

1:800

10Ab - 1:800

Primary Ab: 1 - 800 dilution

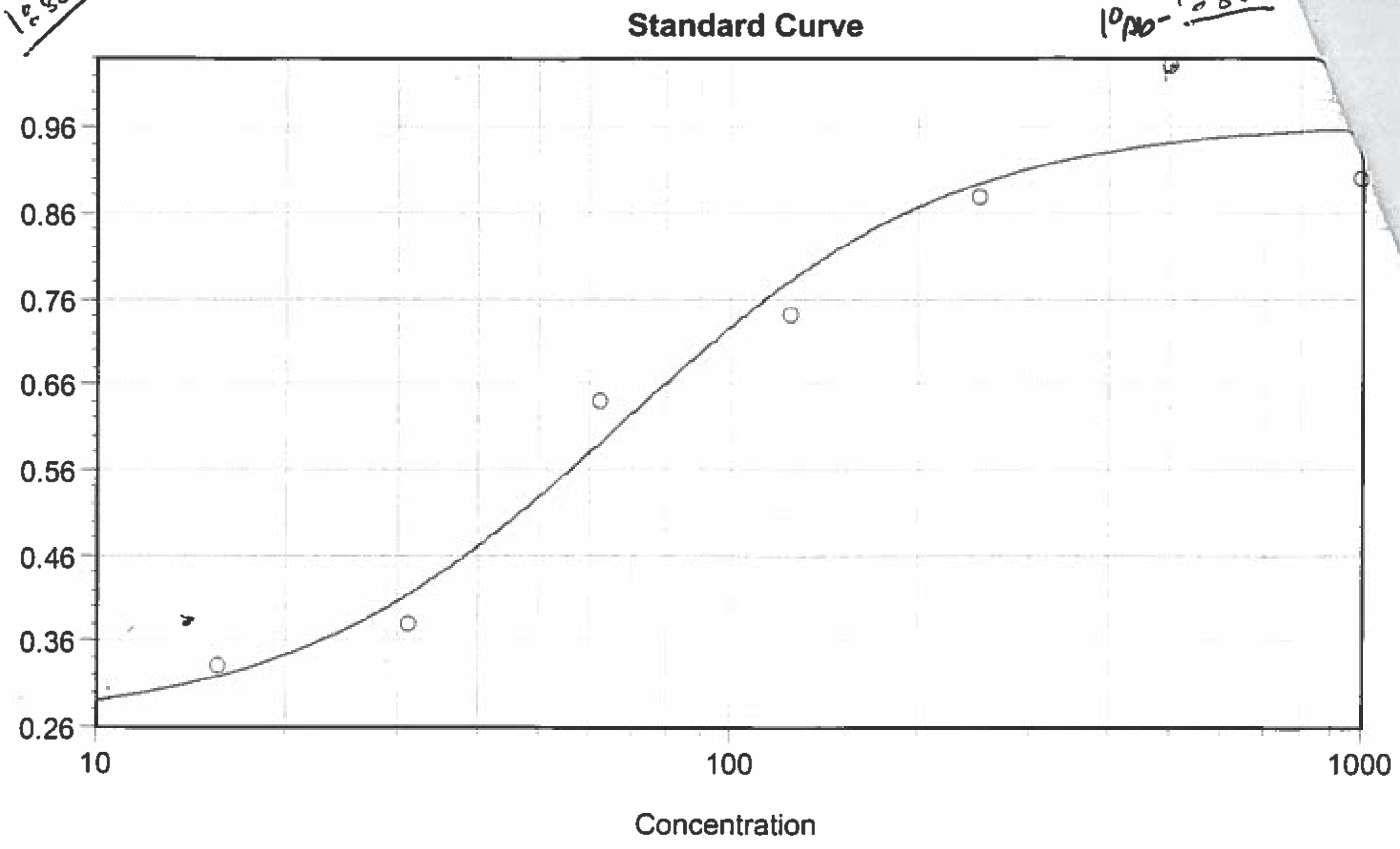

$y = ((A - D)/(1 + (x/C)^B)) + D$:

	A	B	C	D	R^2
o Std (Standards: Concentration vs MeanValue)	0.263	1.674	67.689	0.965	0.971

Experiment#2

CuvetteSet#1

A1 Sa01	A2 Sa02	A3 Sa03	Endpoint
Data: No Data<br>Ref: No Reference	Data: No Data<br>Ref: No Reference	Data: No Data<br>Ref: No Reference	Lm1 260<br>Lm2 280

Wavelength Combination: !Lm1
Data Mode: Absorbance

Samples

Sample	Wells	Sample#	OD260	OD280
Sa01	A1	1		
Sa02	A2	2		
Sa03	A3	3		

Copyright © 2004 Molecular Devices. All rights reserved.

(Ser#01 [illegible])

Experiment#1

1^0 Ab → 1:1000

4- parametric- plot.

Standard Curve

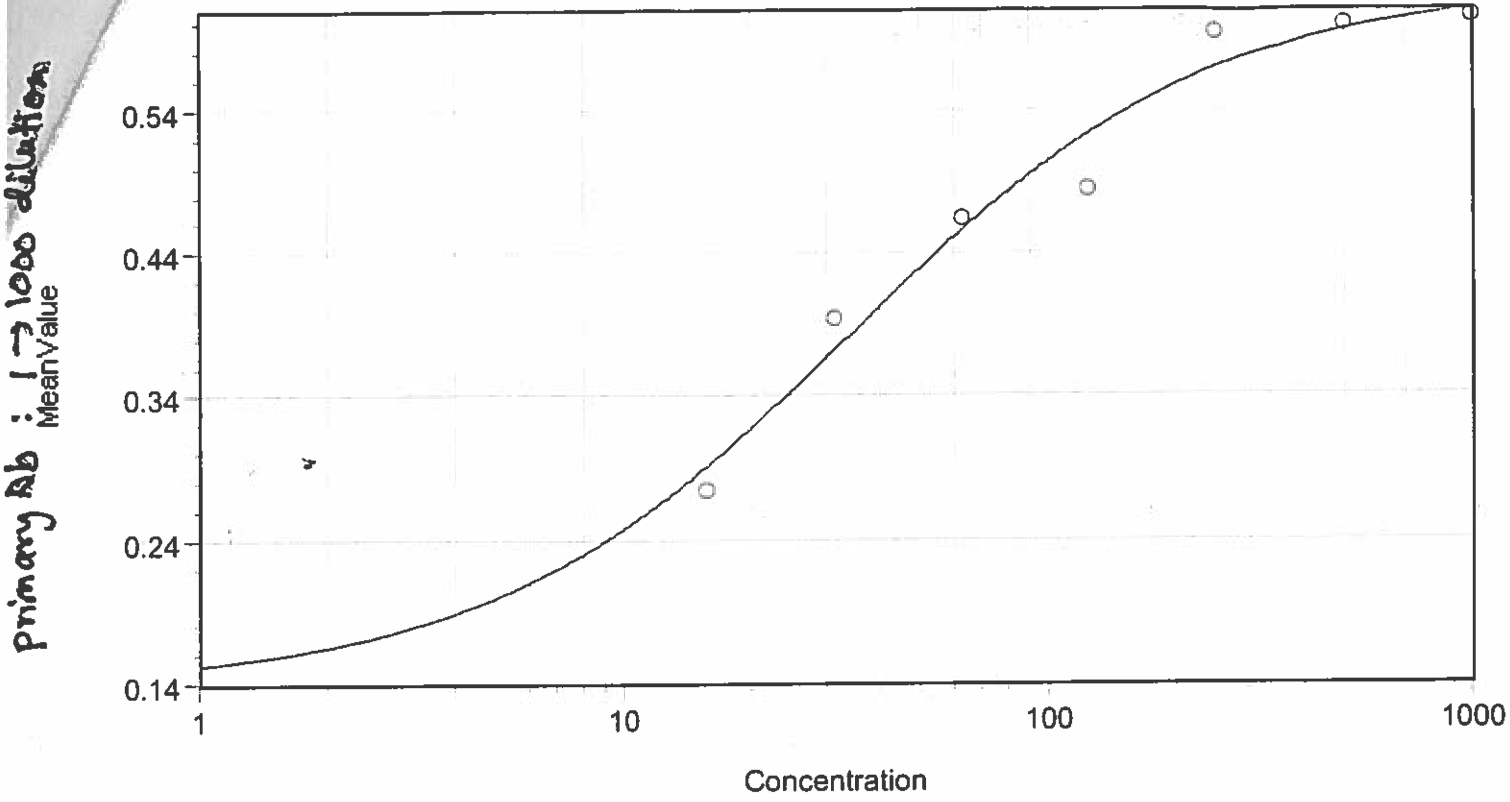

y = ((A - D)/(1 + (x/C)^B)) + D:	A	B	C	D	R^2
o Std (Standards: Concentration vs MeanValue)	0.14	1.025	33.745	0.624	0.985

Copyright © 2004 Molecular Devices. All rights reserved.

UnknownsDilution

Sample	Wells	Values	R	Result	MeanResult	Std.Dev.	CV%	Dilution	Adj.Result

R - Outside standard range
Mean Adjusted Result:

1° Ab = 1:600
2° Ab = 1:10,000.

Control

Sample	Wells	Sample#	Values	MeanValue

Standard Curve

2° Ab : 1 → 10,000 dilution

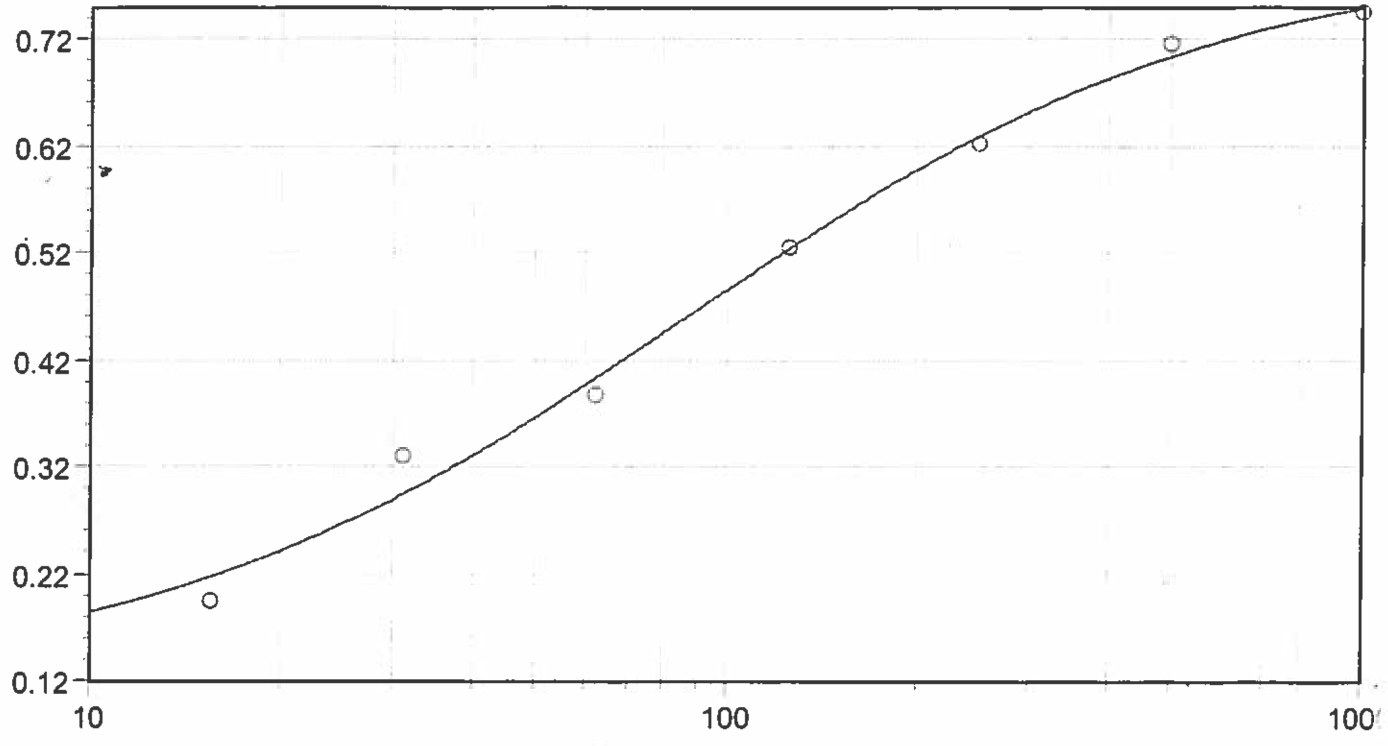

$y = ((A - D)/(1 + (x/C)^B)) + D$:

	A	B	C	D	R^2
o Std (Standards: Concentration vs MeanValue)	0.118	1.035	85.725	0.799	0.994

Experiment#2

CuvetteSet#1

A1 Sa01	A2 Sa02	A3 Sa03	Endpoint
Data: No Data	Data: No Data	Data: No Data	Lm1 260
Ref: No Reference	Ref: No Reference	Ref: No Reference	Lm2 280

8/26/2016 10:17:11 AM 0 color 8252016 set1 600 readout.p

Copyright © 2004 Molecular Devices. All rights reserved.

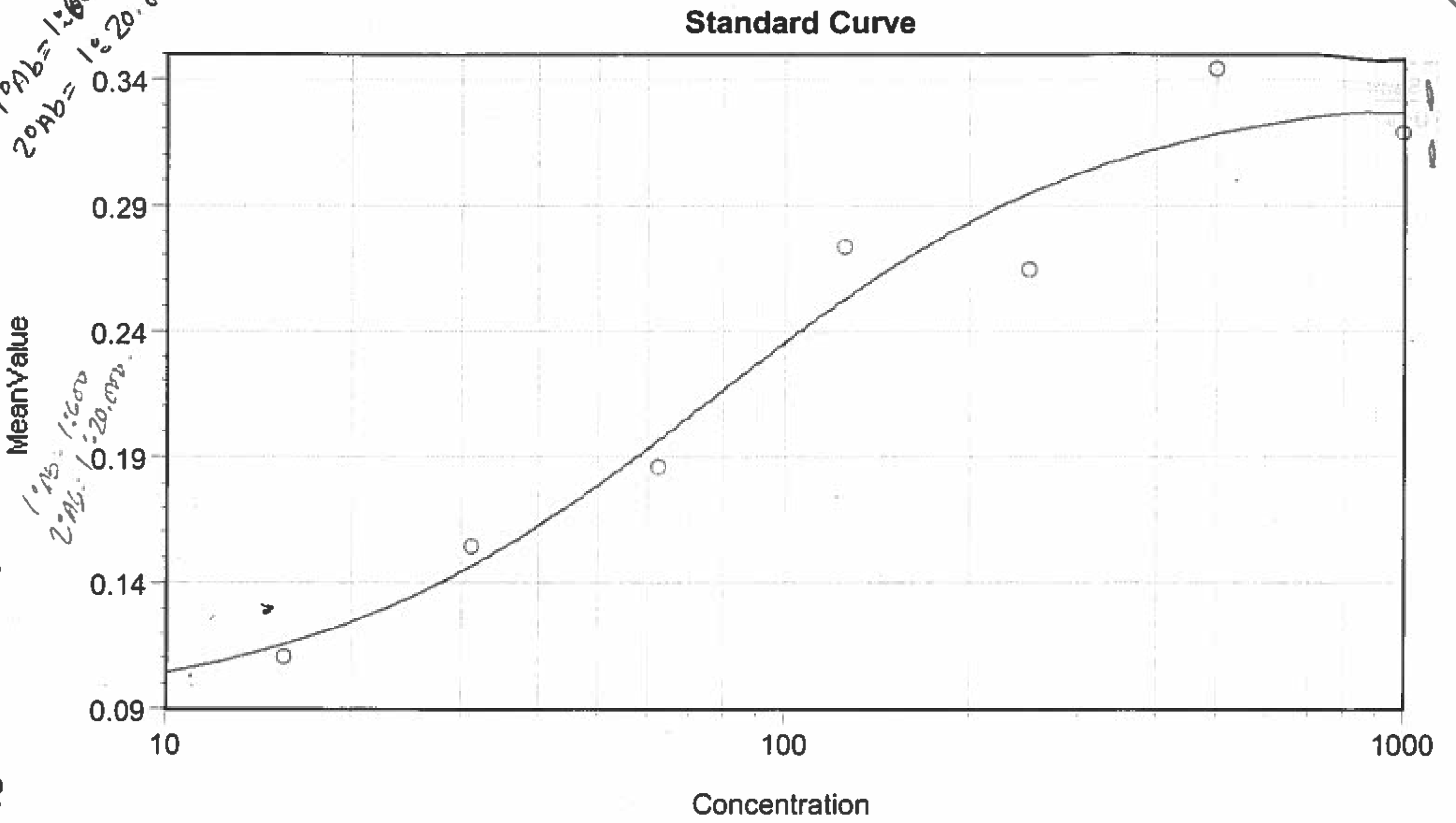

$y = ((A - D)/(1 + (x/C)^B)) + D$:

	A	B	C	D	R^2
○ Std (Standards: Concentration vs MeanValue)	0.09	1.349	76.193	0.336	0.964

Experiment#2

CuvetteSet#1

A1 Sa01	A2 Sa02	A3 Sa03	Endpoint
Data: No Data<br>Ref: No Reference	Data: No Data<br>Ref: No Reference	Data: No Data<br>Ref: No Reference	Lm1 260<br>Lm2 280

Wavelength Combination: !Lm1
Data Mode: Absorbance

Samples

Sample	Wells	Sample#	OD260	OD280
Sa01	A1	1		
Sa02	A2	2		
Sa03	A3	3		

Copyright © 2004 Molecular Devices. All rights reserved.

1° - 600
2° - 20,000 + 2.5% BSA

2° Ab: 1→20,000 + 2.5% BSA.

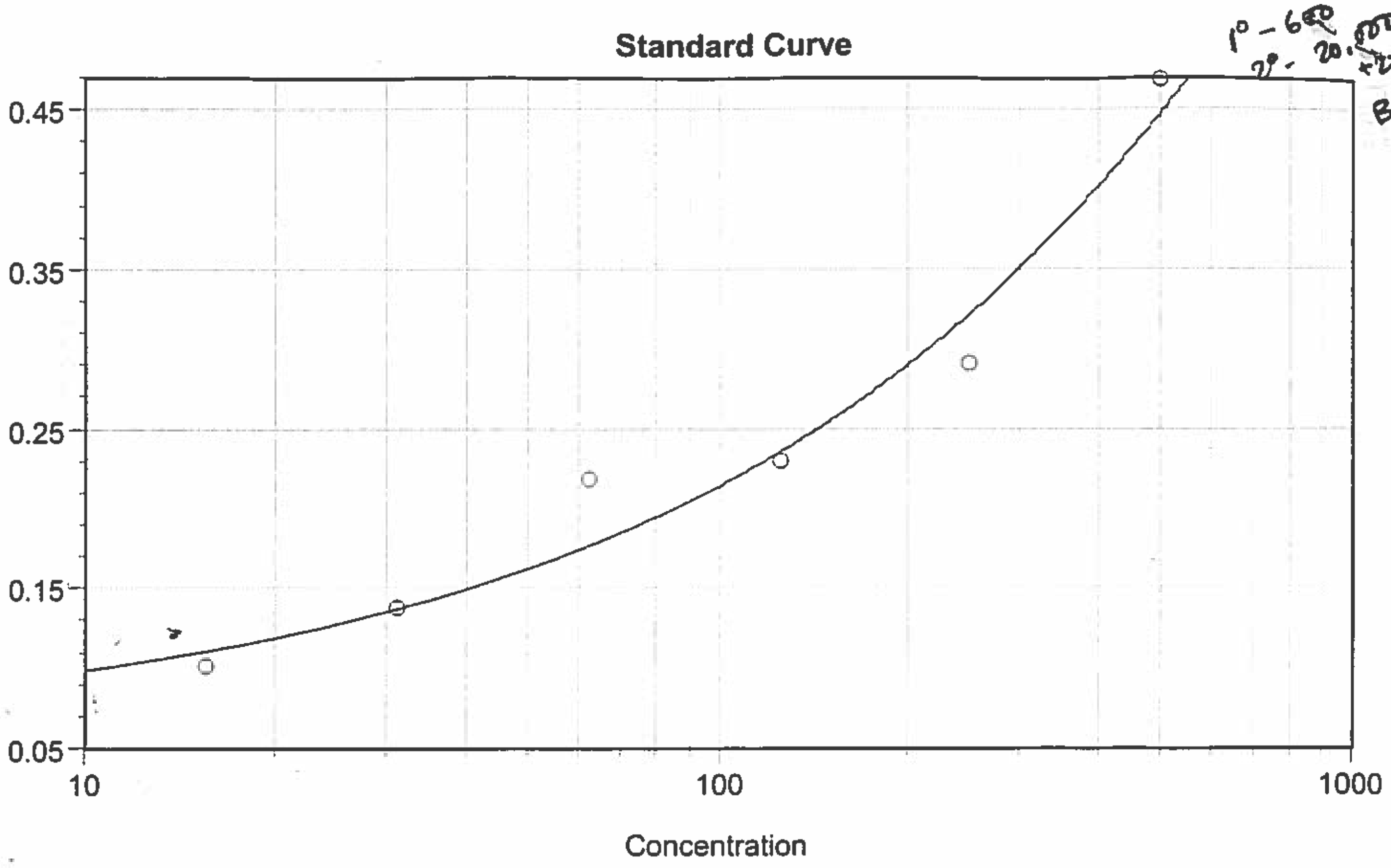

y = ((A - D)/(1 + (x/C)^B)) + D:	A	B	C	D	R^2
o Std (Standards: Concentration vs MeanValue)	0.06	0.613	21294.52	4.319	0.972

Experiment#2

CuvetteSet#1

A1 Sa01	A2 Sa02	A3 Sa03	Endpoint
Data: No Data	Data: No Data	Data: No Data	Lm1 260
Ref: No Reference	Ref: No Reference	Ref: No Reference	Lm2 280

Wavelength Combination: !Lm1
Data Mode: Absorbance

Samples

Sample	Wells	Sample#	OD260	OD280
Sa01	A1	1		
Sa02	A2	2		
Sa03	A3	3		

Copyright © 2004 Molecular Devices. All rights reserved.

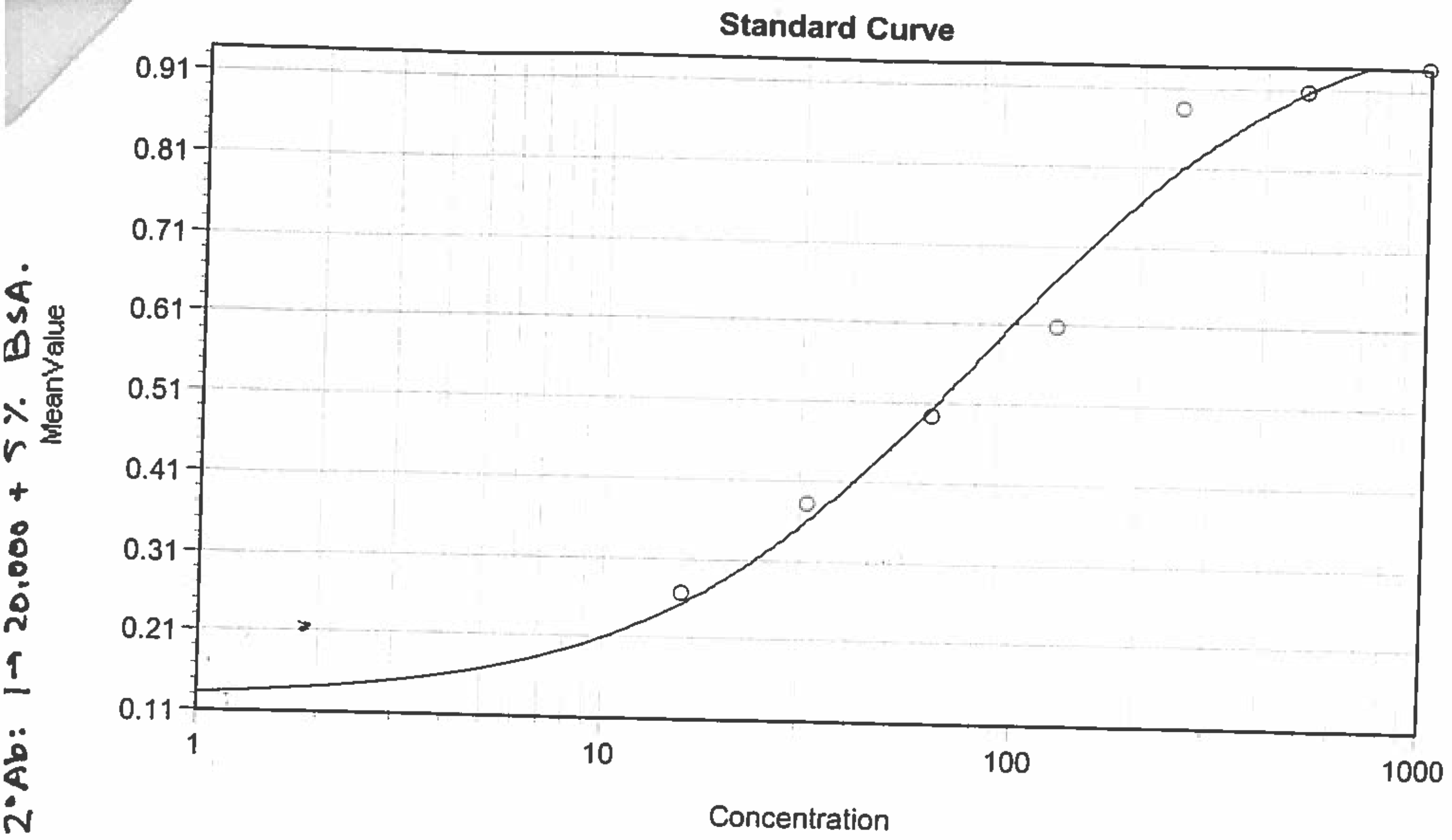

4-P Fit: y = (A - D)/(1 + (x/C)^B) + D:	A	B	C	D	R^2
o Std (Standards: Concentration vs MeanValue)	0.124	1.06	83.7	1.02	0.984

Weighting: Fixed

Pearson correlation
correlate
↓
Bivariate
↓
Pearson
↓
variables
OK
